# The Unique Immunomodulatory Properties of MSC-Derived Exosomes in Organ Transplantation

**DOI:** 10.3389/fimmu.2021.659621

**Published:** 2021-04-06

**Authors:** Qingyuan Zheng, Shuijun Zhang, Wen-Zhi Guo, Xiao-Kang Li

**Affiliations:** ^1^Department of Hepatobiliary and Pancreatic Surgery, The First Affiliated Hospital of Zhengzhou University, Zhengzhou, China; ^2^Division of Transplantation Immunology, National Research Institute for Child Health and Development, Tokyo, Japan

**Keywords:** immunomodulation, MSC-derived exosomes, immune tolerance, transplantation, GvHD

## Abstract

Methods for suppressing the host immune system over the long term and improving transplantation tolerance remain a primary issue in organ transplantation. Cell therapy is an emerging therapeutic strategy for immunomodulation after transplantation. Mesenchymal stem cells (MSCs) are adult multipotent stem cells with wide differentiation potential and immunosuppressive properties, which are mostly used in regenerative medicine and immunomodulation. In addition, emerging research suggests that MSC-derived exosomes have the same therapeutic effects as MSCs in many diseases, while avoiding many of the risks associated with cell transplantation. Their unique immunomodulatory properties are particularly important in the immune system-overactive graft environment. In this paper, we review the effects of MSC-derived exosomes in the immune regulation mechanism after organ transplantation and graft-versus-host disease (GvHD) from various perspectives, including immunosuppression, influencing factors, anti-inflammatory properties, mediation of tissue repair and regeneration, and the induction of immune tolerance. At present, the great potential of MSC-derived exosomes in immunotherapy has attracted a great deal of attention. Furthermore, we discuss the latest insights on MSC-derived exosomes in organ transplantation and GvHD, especially its commercial production concepts, which aim to provide new strategies for improving the prognosis of organ transplantation patients.

## Introduction

Organ transplantation is the most effective treatment for patients with end-stage disease, and it is one of the most noticeable and important achievements in the development of medicine in the 20th century (1). Although the short-term survival rate of allogeneic organ transplantation has been greatly improved, the development of methods to increase the long-term survival of transplanted organs remains a major challenge in clinical medicine. Currently, the combined application of immunosuppressant is the main method to prevent anti-rejection reaction after organ transplantation, but it is not sensitive to chronic rejection (2). Thus, the potential application of cell therapy in improving recipient tolerance to transplantation has attracted widespread interest.

Mesenchymal stem cells (MSCs) are a group of self-renewing stem cells with broad differentiation potential, implantation and homing capabilities, and immunomodulatory effects; thus, they have been increasingly applied in clinical studies (3–5). MSCs derived from the development of early mesoderm and ectoderm, showed the expression of major histocompatibility complex I (MHC-I), CD90, CD105, and CD73, but did not express CD45, CD34, CD14, or CD11b (6). Under certain circumstances, they can be induced to differentiate into connective tissue, bone, cartilage, fat and bone marrow stromal cells. Some studies have confirmed that MSCs had an immunosuppressive effect, which can play an immunomodulatory role by inhibiting T cell proliferation, preventing B cell activation, affecting the differentiation, maturation, and function of dendritic cells (DCs) and interfering with the activation and maturation of antigen-presenting cells (APCs) (7–10). Besides, a study found that MSCs may inhibit IL-2-induced NK cell activity *in vitro* (11). Studies have shown that the paracrine function of MSCs is the key mechanism for the immune function. Exosomes are important molecules that play a role in the transmission of information and biological functions. This review systematically summarizes the mechanism, influencing factors and clinical applications of MSC immunosuppression, especially the research and prospects of MSC-derived exosomes in the field of organ transplantation and graft-versus-host disease (GvHD).

## The Immunosuppressive Effect of MSCs and Its Mechanism

**Figure 1:**
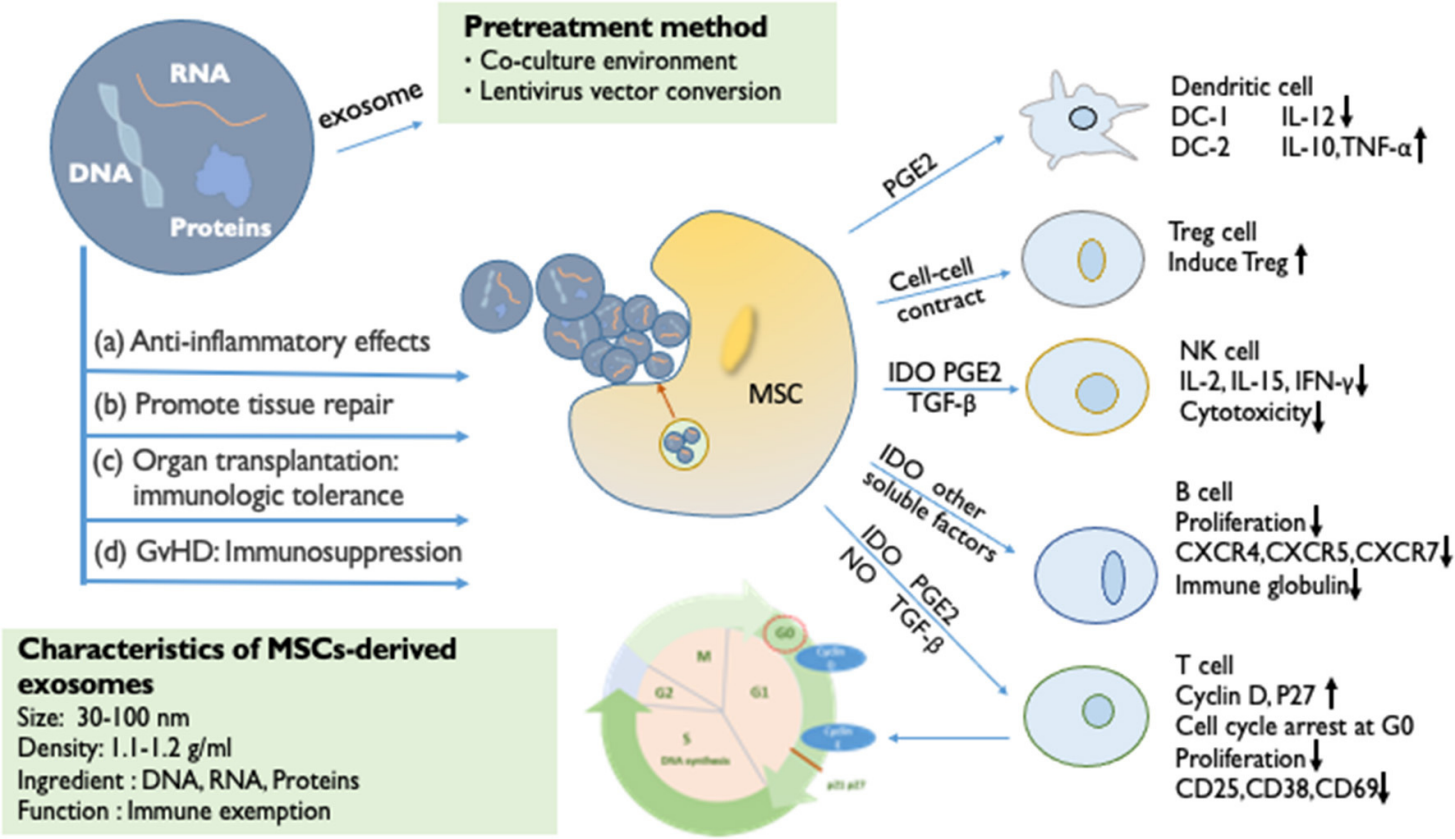
The immunosuppressive effect of MSCs and MSC-derived exosomes. The immunomodulatory effects of MSCs can be largely attributed to paracrine factors (IDO, PGE2, NO, TGF-β, and exosome). By secreting soluble factors, the proliferation of T cells is inhibited (cell cycle arrest at G0), B cell activation is prevented (reduced proliferative capacity), NK cells are influenced (reduced cytotoxicity), and the differentiation and maturation of DC is affected. Through cell-to-cell contact, it can Tregs can be induced. MSC-derived exosomes are small vesicles of 30–100 nm in diameter, which are rich in important biomolecules such as DNA, RNA, and proteins. MSC-derived exosomes have the same immunosuppressive effect as MSCs. (a) Anti-inflammatory effects. (b) Promotion of tissue repair. (c) Organ transplantation: immunologic tolerance. (d) Immunosuppression in GvHD. The targeted pretreatment of exosomes is an important means of current biological treatment. The method of changing the co-culture environment, and the lentiviral vector transferring functional proteins or mRNA into exosomes, can pre-enhance the activity of exosomes to enhance efficacy.

MSCs have an immunomodulatory effect that influences all cells involved in the immune response (Figure 1). MSCs inhibit T cell activity, induce regulatory T cell activation, trigger cell apoptosis and arrest the cell cycle in the G0/G1 phase. Glennie et al. (12) found that the potential for the inhibition of B cell proliferation by MSCs is similar to that of T cells. It has been confirmed that MSCs can inhibit the proliferation of natural killer (NK) cells and interferon IFN-γ stimulated by IL-2 or IL-15(13). In summary, the MSCs inhibit the immune responses and effector cells of the memory cell, thereby modulating innate immunity and adaptive immunity. Furthermore, MSCs can increase the amounts of regulatory T cells (Tregs), regulatory B cells (Bregs), and regulatory DCs (DCregs)—resulting in the loss of function of T cells—to regulate the immune function (14). BM-MSCs have also been proven to inhibit the proliferation and cytotoxicity of NK cells by changing the phenotype of NK cells and cell-to-cell contact (15, 16). In general, MSCs produce resistant cytokines, which directly inhibit the ongoing immune response and inhibit the proliferation of lymphocytes. Circulating hematopoietic cells actively home into the wall across the vascular endothelium of different organs and bone marrow; this is important for the host defense and repair functions (17). This depends on multiple molecular signals, including growth factors, chemical factors, and adhesion factors. The plastic adhesion properties of MSCs may have chemotactic functions (18). Similarly to other immune cells at sites of injury and inflammation, with the help of the homing ability, MSCs chemotactically transport to target organs, mediating anti-inflammatory response and tissue repair and regeneration. These functions represent a key feature that plays a role in regenerative medicine.

MSCs have effective immunomodulatory properties and anti-inflammatory abilities, which produce extracellular vesicles, including exosomes, and a large number of cytokines and growth factors. The inhibition of the proliferation of T and B cells and the maturation of monocytes promotes the production of Tregs and M2 macrophages. Studies have shown that when the paracrine mechanism of MSCs is activated, they can directly secrete anti-inflammatory cytokines, such as indolamine 2,3-dioxygenase (IDO), prostaglandin E2 (PGE2), nitric oxide (NO), HLA-G, transforming growth factor-β (TGF-β), and vascular endothelial growth factor (VEGF) (19, 20). These cytokines affect the number of immune cells and achieve the effect of suppressing the immune response. Among them, PGE2 and IDO have a synergistic effect (21). Research by Cho et al. (22) showed that BM-MSCs can convert bone marrow-derived macrophages from M1 to M2, promote the proliferation of M2 macrophages, enhance the ability of macrophages to inhibit inflammation, and weaken their pro-inflammatory ability. The study by Vasandan et al. (23) was consistent with the above findings. MSCs produce soluble factors in response to inflammatory stimuli to induce the activation of the Treg, DCreg, and M2 populations in a variety of inflammatory diseases. Thus, even if the MSCs soon disappear *in vivo*, these cytokines can still suppress the immune level of the body for a long time (24).

According to several *in vivo* and *in vitro* studies, the immunosuppressive effect of MSCs is affected by many factors, such as different cell sources, administration methods and dosages, and the microenvironment created by the interaction with fibroblasts, endothelial cells, epithelial cells, and macrophages. For MSCs from different sources, although their proliferation and differentiation abilities are similar, they may show different specific phenotypes and immunomodulatory properties, resulting in differences in efficacy. Harman et al. (25) performed single-cell RNA sequencing (scRNA-seq) on primary equine MSCs that were collected from adipose tissue, bone marrow, and peripheral blood. They observed that transcriptional differences corresponded with phenotypic variance in cellular motility and the immune regulatory function (25). Meanwhile, the timing of administration, dosage and state of activation of MSCs has a great influence on the *in vivo* efficacy. In general, the dose of MSCs that is intravenously injected in mice is 5 × 10^7^/kg, while the clinical dose for humans is usually 1–2 × 10^6^/kg (26). When BM-MSCs were intravenously administered preoperatively at doses of 0.5 × 10^6^/kg and 1.0 × 10^6^/kg, combined with mycophenolate mofetil (MMF), to a mouse model of allogenic heart transplantation, the graft survival rates were different. Taken together, only the MSC-MMF combination of three different MSC—drug combinations led to a super-additive immunosuppressive effect. And the survival time of grafts in the low-dose group of BM-MSCs was higher (27). A great deal of further experimentation is needed to investigate the dose-dependent effects. The immunosuppressive effects and mechanisms of MSCs derived from different tissues of the same species are also different. With the responder T lymphocyte population in mixed lymphocyte culture (MLC), the allogeneic and autologous MSCs induced a significant differentiation of CD4^+^ T-cell subsets co-expressing CD25. However, only allogeneic MSCs favored an increase in cytotoxic T lymphocyte antigen-4+ populations. With respect to autologous MSCs, allogeneic MSCs may play a more effective suppressive activity (28). In particular, the positioning and migration of MSCs after *in vivo* injection are involved in the long-term tolerance of the graft by secreting soluble factors in close contact with the target site. At present, the combined use of multiple immunosuppressive agents causes different degrees of immunodeficiency, causing side effects, such as infection and malignant tumors, which are major problems after organ transplantation. However, whether MSCs can be used in combination with immunosuppressive agents remains to be verified. Will MSCs permanently alter the body's immune response mechanisms? We hypothesize that in a complex *in vivo* microenvironment, MSCs promote long-term tolerance of the graft and will also induce rejection. Therefore, an in-depth understanding of the interaction between MSCs and other cells is particularly important for the improvement of stem cell therapy in the future.

## MSC-Derived Exosomes

Exosomes were first discovered in 1983 in the reticulocyte network. They are considered to be a physiological cystic structure of waste from the process of red blood cell development. They remove specific proteins during the maturation of reticulocytes. Exosomes includes DNA, mRNA, miRNA, and proteins and other important molecules. Active exosomes are secreted by a variety of cells of 30–100 nm in diameter, which have a lipid bilayer structure, and the distribution of Four transmembrane protein superfamilies; they carry and transport proteins, nucleic acids, lipids and other biologically active molecules (Figure 1) (29–32). In the process of transportation, exosomes not only maintain their biological activity but also can distribution of the transmembrane protein modify and process molecules, which plays an important role in the transduction of intercellular material and information (33). MSCs can tend to the injured site and perform the functions of tissue repair, wound healing, and immune regulation. Immunization exemptions are one reason for their high-profile in the field of organ transplantation (34). The paracrine effect is one of the important mechanisms for MSCs (35, 36). Bruno et al. (37) revealed that exosomes play an important role in the paracrine effect of MSCs. MSC-derived exosomes with cytokines and growth factors, lipid signals, mRNA, and miRNA regulatory biomolecules have a biological function or information transfer function (38, 39). They have similar functions to MSCs, including the promotion of tissue repair, immunosuppression, and neuroprotection. As a medium component of cell-to-cell communication, exosomes spread biological information between cells in the form of paracrine and remote secretion, and play an important regulatory role in physiological and pathological conditions.

More and more studies have shown that MSC-derived exosomes have the ability to affect the activity of immune cells, including T cells, B cells, NK cells, and macrophages. Studies have suggested that the therapeutic effect of exosomes is mediated by mRNA delivery. Many of these are involved in transcription regulation, proliferation, and immune regulation, indicating that they contribute to the induction of tissue regeneration (40). In addition, the function of exosomes is also affected by other molecules (such as proteins, lipids, enzymes, signal molecules) to exert their functional activities. These proteins are mainly involved in cell adhesion, membrane fusion and signal transduction (41). Those most common proteins are tetraspanin and integrin proteins (CD63, CD9, CD81, and CD82), which is crucial for cell adhesion. They are located on the surface of exosomes as markers (42, 43). Furthermore, the proteins involved in membrane fusion are Rab GTPases, annexins, and heat shock protein (HSP70 and HSP90) (43). Exosomes can interact with recipient cells by ligand-receptor binding or direct endocytosis. Exosome uptake by recipient cells is cell-specific. The interaction of surface molecules of exosomes is important for recipient cell targeting (44, 45). However, the complex microenvironment between different molecules or different cells causes the exosomes derived from MSC to be not equivalent. Exosomes of the same cell express different epitopes, indicating that there may be different subtypes of exosomes. Compared with BMSC-derived exosomes, AD-MSC contain up to 4-fold higher levels of enzymatically active neprilysin (24, 46). The molecules that synthesize exosomes actually depend on the cell type. MSCs are a major component of the tumor microenvironment (TME). MSC-derived exosomes will be altered when MSCs are cultured in TME. MSCs remodel the extracellular matrix, and tumor-derived small vesicles may exert profound effects on tumor growth (47, 48). Hypoxia injury is one of the main causes of apoptosis and decreased β-cell function in islet grafts. In organ transplantation, a study found that huMSC-derived exosomes played an important role in hypoxic resistance, which could protect neonatal porcine islet cell clusters from hypoxia-induced dysfunction (49). The functions of different microenvironments are complex and changeable. In conclusion, we should further explore the interaction between different molecules on the surface or within exosomes.

## Anti-inflammatory and Tissue Repair Promoting Effects of MSC-Derived Exosomes

Recent studies have shown that MSCs will not directly implant and replace damaged tissues, and the exosomes secreted by MSCs are considered to be key cytokines with strong anti-inflammatory potential. A clinical study showed that MSC-derived exosomes may reduce the ability of peripheral blood mononuclear cells (PBMCs) to release proinflammatory cytokines *in vivo*. MSC-derived exosomes upregulated IL-10 and TGF-β1 from PBMCs, thereby promoting the proliferation and immunosuppression capacity of Tregs (50). Another study found that MSC-derived exosomes can reduce the neuroinflammation of autoimmune encephalomyelitis in mice and increase the number of Tregs to generate innate immunity (51). MSCs have been used for treating the inflammatory storm caused by severe pneumonia in COVID-19 patients, MSC-derived exosomes are more convenient and superior, and are recommended for alternative treatments (52).

The exosomes obtained after IFN-γ stimulation reduce the proliferation of PBMCs *in vitro*, reduce the pro-inflammatory factors and increase the immunosuppressive factor IDO (53, 54). In a study involving traumatic brain injury, Long et al. found that MSC-derived exosomes reduce the level of pro-inflammatory cytokines (55). Exosomes have been used as a treatment to reduce neuroinflammation after brain injury (56). Besides, MSC-derived exosomes have a special effect on the treatment of necrotizing enterocolitis (57–59) and bronchopulmonary dysplasia (60, 61). In a mouse model of bronchoalveolar dysplasia, MSC-derived exosomes can regulate the changes in the M1/M2 phenotype of macrophages to reduce the inflammatory damage caused by hyperoxia (62). Studies have found that Human Wharton's jelly mesenchymal stem cell (hWJ-MSC)-derived exosomes have anti-inflammatory effects in perinatal brain injury (63). We therefore hope to explore the anti-inflammatory potential of exosomes derived from hWJ-MSCs in the treatment of brain injury. *In vitro*, exosomes affected microglia and reduced the LPS-stimulated release of pro-inflammatory cytokines. *In vivo*, the intranasal administration of hWJ-MSC-derived exosomes is an effective method for reducing neuroinflammation.

MSC-derived exosomes are small in size, and among the non-coding RNAs (miRNA and LncRNA) involved in immunosuppressive effects, the shorter miRNA is mostly involved in mRNA post-transcriptional regulation of gene expression levels, and thereby controls the key pathways of cell development and differentiation (64). Studies have found that in the brain damage caused by stroke, the increase of miR-133b in exosomes released by bone marrow mesenchymal stem cells may be realized by the transfer of exosomes from MSCs to the parenchyma (65, 66). miR-133b transferred through the exosomes from MSCs into astrocytes may downregulate the expression of connective tissue growth factor (CTGF), reduce glial scarring, and facilitate axonal growth. Meanwhile, miR-133 downregulates the expression of RhoA protein, while inhibiting RhoA promotes the regeneration of the corticospinal tract after spinal cord injury (67). These studies have shown that MSC-derived exosomes secrete mRNA to induce the regeneration of damaged tissues.

In an animal study of acute myocardial infarction, ligation of the left anterior descending artery (LAD) in rats induced cardiomyocyte apoptosis, and the immediate intravenous injection of human umbilical cord mesenchymal stem cells (hucMSC)-derived exosomes (400 μg of protein) could significantly improve cardiac contractility and reduce heart fibrosis. They hypothesized that hucMSC-derived exosomes may protect cardiomyocytes from apoptosis by regulating the expression of the Bcl-2 family and promote the tube formation and migration of vascular endothelial cells (68). Exosomes secreted by hucMSCs protect cardiomyocytes from anoxia-reoxygenation injury (69). Similar studies are equally effective in mouse ischemia/reperfusion injury models (70). hucMSC-derived exosomes can alleviate CCl_4_-induced liver fibrosis, prevent cisplatin-induced renal oxidative stress and cell apoptosis, and enhance skin wound healing.

## Research and Exploration of MSC-Derived Exosomes in the Field of Organ Transplantation

Pre-infusion of different regulatory cells (including MSCs, Tregs and DCs) is currently a viable alternative therapy for transplant recipients. At present, the clinical application of MSCs in organ transplantation used to improve the prognosis of transplant recipients has broad prospects. However, efficiency is still the biggest concern in clinical research. Most clinical-stage MSC therapies have been unable to meet primary efficacy endpoints. This creates issues with practicality and feasibility (71). Although MSC-derived exosomes have similar functions to MSCs, the direct application of MSC-derived exosomes is not yet mature. Studies have proven the alloantigen presentation and immune regulation abilities of exosomes, which induced immune tolerance in a rat allograft model (72).

In allotransplantation, MHC antigen is the main foreign antigen that induces transplant rejection (73). The allograft survival rate mainly depends on the degree of human leukocyte antigen (HLA) type matching between the recipient and donor. Exosomes are MHC-bearing vesicles that are secreted by various cells. A study from the Institute of Transplantation of the University of Nantes in France found that exosomes express MHC-I and MHC-II (74, 75). The researchers found that injecting donor DC-derived exosomes both prolonged the survival time of rat heart allografts before and after transplantation. Interestingly, two injections of donor DC-derived exosomes at 2 and 1 week, respectively, prior to heart allotransplantation did not induce tolerance to long-term graft survival. Thus, the researchers used LF15-0195 (a new type of immunosuppressant) with exosomes for short-term combination therapy, and found that it effectively prevented the maturation of DCs. Exosome/LF treatment prevented or significantly delayed the appearance of chronic rejection. MHC antigens from donor exosomes strongly suppressed the anti-donor proliferation response. Coincidentally, combined with rapamycin, donor exosomes from immature dendritic cells (imDex) can prolong the survival of cardiac allografts and induce specific allograft tolerance (76). These results suggest that the presentation of donor MHC antigens (from exosomes) in combination with immunosuppressive therapy induces a regulatory response that modulates allograft rejection and induces donor-specific allograft tolerance. It is noteworthy that data showed that the number of damaged blood vessels was significantly reduced in 60% of transplanted rats and that exosomes completely prevented chronic rejection in 40% of cases at 200 days after transplantation (74).

Immunosuppressants are the main treatment for avoiding immune rejection after organ transplantation. Exosomes combined with suboptimal doses of immunosuppressants may achieve specific allograft tolerance and long-term transplant survival (72). It is worth noting that it is difficult to achieve tolerance or long-term survival without the use of immunosuppressants. In a rat intestinal transplantation model, the intravenous infusion of exosomes from imDex (20 μg) before transplantation could reduce the host's anti-donor cell response, induce the production of Tregs, and prolong the survival of allografts (77). The long-term use of immunosuppressants can increase the risk of infection, and it is important to determine the optimal dose or find alternative therapy. Tregs can protect allografts from immune rejection. Ma et al. (78) found that imDex combined with Tregs could induce immune tolerance in a rat liver transplantation model. Post-transplant liver samples were obtained for HE staining by researchers at 0, 10, 35, and 100 days after transplantation. Interestingly, the imDex and Tregs treatment groups, respectively, showed a high number of inflammatory infiltrates and symptoms of chronic rejection (e.g., biliary atresia and cholestasis). However, there were no symptoms of chronic rejection in the co-processing group. At 100 days, the co-processing group of grafts showed regenerated hepatic fibrous tissue. Although the structure of the hepatic lobules is disordered, mononuclear cell infiltration is reduced. After liver transplantation, chronic rejection leads to structural disorder of hepatic lobules and tissue fibrosis. The immunomodulatory effects of MSC-derived exosomes and their promotion of angiogenesis and tissue repair effectively alleviated the progress of the pathological process. Thus, we confirm that MSC-derived exosomes can be an important foundation for the treatment of chronic rejection of the field of liver transplantation. Co-treatment reduced rejection and helped the recipient liver regenerate after undergoing slight acute rejection (78).

During transplantation, graft injury caused by cold ischemia, organ preservation, and reperfusion has always been a matter of concern. After ischemic injury, the graft will undergo a second blow of reperfusion injury as blood flow opens up. In an experiment using a rat model of kidney donation after circulatory death, the researchers used Belzer solution (BS) and BS supplemented with MSC-derived exosomes to compare renal perfusion injury. During cold organ perfusion (4 h), the signs of kidney damage were significantly less severe in DCD kidneys treated with MSC-derived exosomes (79). Ischemia reperfusion injury (IRI) is an important cause of liver failure after liver resection and early non-immunological inactivation after liver transplantation. It also increases the chance of acute and chronic rejection of the transplanted organ and leads to late immunological inactivation (80). Preconditioning of graft perfusion with MSCs and MSC-derived exosomes before transplantation may be an effective method for limiting ischemia-reperfusion injury. Using a normothermic hypoxic rat liver perfusion model, Rigo et al. (81) perfused the graft for 4 h and delivered extracellular vesicles derived from human hepatic stem-like cells (HLSCs), and found that the levels of hepatocyte damage and hepatocyte lysis markers in the lavage fluid were reduced. This effect has also been verified in the lung (82) and heart (83). Although they did not use MSC-derived exosomes, we could also observe that using exosomes would be very likely to limit the graft damage caused by ischemia-reperfusion before and after transplantation (84–87).

In addition, in heart transplantation, a study found a new biomarker method in which donor heart exosomal signals are monitored to understand the intensity of immune rejection after transplantation. We can use exosomes from the recipient's blood or urine for functional monitoring of allograft dysfunction and rejection. This is an effective way to reduce the pain of invasive biopsies (88). Studies have confirmed that exosomes can be used as biomarkers for tolerance monitoring in kidney (89), heart (90), liver (91), islet (92), and lung (93) transplantation. Furthermore, in post-myocardial infarction inflammation, hucMSC-derived exosomes increased the density of infarct myofibroblasts, reduced inflammation, and promoted the differentiation of fibroblasts into myofibroblasts inflammation *in vitro*. Researchers hypothesize that human MSC-derived exosomes may have a cardioprotective effect. MSCs-secretions improve the donor heart function following *ex vivo* cold storage (94). In a porcine model of myocardial infarction subjected to intravenous bolus injection, myocardial infarction measured at 7 and 28 days significantly reduced the infarct size (30–40%) (95). This opens up new ideas for fibrosis caused by chronic rejection after organ transplantation. Jiang et al. (70) also found MSC-derived exosomes can reduce oxidative stress, the inhibition of hepatic apoptosis, and reduced CCl_4_-induced liver fibrosis. In engrafted liver mouse models, a single systemic administration of human MSC-derived exosomes (16 mg/kg) effectively rescued the recipient mice from CCl_4_-induced liver failure (96). IDO-BMSC-derived exosomes can be used to improve immune tolerance and prolong survival of heart allotransplantation (97). Given the few existing studies on MSC-derived exosomes in the transplantation field, this will be a new research direction. Combined with the previous statement, MSC-derived exosomes are beneficial for tolerance induction in organ transplantation. Considering its characteristics as a drug carrier, the use of exosomes will undoubtedly become a direction of scientific and technological development in the field of organ transplantation (98).

Furthermore, in numerous studies exploring the roles of exosomes in tumor immunity, exosomes have a dual function of adjustment on tumor cell proliferation (99, 100). Following the injection of MSC-derived exosomes into a mouse model of liver cancer, the anti-tumor miR-122 and other secreted biological molecules not only effectively inhibited tumor growth, but also significantly increased the antitumor efficacy of sorafenib against hepatocellular carcinoma (101). Another study found that MiR-199a-modified exosomes from AD-MSCs improved hepatocellular carcinoma chemosensitivity through the mTOR pathway (102). Multiple studies have shown that exosomes combined with immunosuppressants or targeted chemotherapeutic agents are effective in the treatment of chronic rejection or cancer. However, the effect of direct infusion of miRNA is easy to degrade. The author believes that MSC-derived exosomes can be used as carriers for drug and molecular delivery, and that pretreatment of target effect miRNA can become a consensus for disease treatment. If pretreated exosomes are used to enhance the sensitivity of chemotherapeutic agents in reduced-time treatment before liver transplantation for patients with liver cancer, it will effectively reduce the perioperative time and improve the patient prognosis. These studies have provided new ideas for the adjuvant treatment of liver cancer patients with liver transplantation (101, 102).

After reviewing the literature, we retrieved 93 studies of exosomes on www.clinicaltrials.gov, including 3 MSC-derived exosomes clinical trials involving acute ischemic stroke and ophthalmic diseases (103). They have also been reported to have therapeutic effects on acute and chronic kidney disease in animal models. Many efforts have been made to prove that MSC and MSC-derived exosomes can produce similar therapeutic benefits in various disease models (24). Clinical application showed the feasibility of exosomes. Current research focuses on preconditioning the graft before transplantation and preventing ischemia/reperfusion injury to improve the viability of transplanted organs (104, 105). In large animal experiments, we can better understand the biological and functional characteristics of MSC-derived exosomes and define the acceptance in allotransplantation. However, there is no studies have been conducted on the application of MSC-derived exosomes in large animal transplantation models. In pig models of traumatic brain injury and hemorrhagic shock, early treatment with a single dose of exosomes provided neuroprotective effects and improved Blood brain barrier integrity (106–108). In fact, using large animals (pigs or monkeys) is more convincing than small animal models. The advantages of large animal experiments include the complexity of the disease, effective cell dose, cell survival rate after transplantation, and tissue inflammation and immune response associated with transplantation (109, 110). Therefore, we need to invest a lot of energy to study the role of MSC-derived exosomes in large animal transplantation models before clinical application. Xenotransplantation is the development direction of organ transplantation (111). However, there is no application of MSC-derived exosomes in xenotransplantation. If MSC-derived exosomes can reduce the risk of rejection of xenotransplantation, it will be a milestone breakthrough. We boldly speculate that with the continuous development of research, the application of MSC-derived exosomes in organ transplantation will produce better and better results.

## MSC-Derived Exosomes and GvHD

MSCs were introduced as a treatment strategy for acute GvHD by Le Blanc et al. (112). The application of MSCs in the treatment of GvHD is summarized in Table 1 (118). As described above, MSC-derived exosomes play the same role as MSCs. In GvHD, a study published in 2014, which describes the first human case in which MSC-derived exosomes were successfully used to treat graft-versus-host disease, seems to indicate that it is feasible to use MSCs as a therapeutic agent for the alternative therapy of various diseases (119). Lai et al. (120) injected MSC-derived exosomes into a GvHD model; this significantly inhibited the activity of Th17 and Treg cells, and reduced immune rejection and pathological damage. MSC-derived exosomes effectively prolonged the survival of chronic GvHD mice and diminished the clinical and pathological scores of chronic GvHD (120). In the same study, in a GvHD model induced by the injection of human peripheral blood mononuclear cells into irradiated mice, MSC-derived exosomes could alleviate the symptoms of GvHD and improve the survival rate. MSC-derived exosomes promoted the production of Tregs *in vivo* and *in vitro* through APC-mediated pathways (121). Interestingly, in the mouse GvHD model, human BM-MSCs were injected intravenously into the mouse 3 days after the operation. When the BM-MSCs undergo macrophage phagocytosis, inflammatory M1 macrophages were functionally attenuated with a concomitant shift toward alternatively activated M2 state. The effector mechanisms of immunosuppression are activated in BM-MSCs, increasing the metabolic conversion efficiency of macrophages and resulting in a large reduction in IDO spleen and lung inflammatory infiltration (23). MSC paracrine factors can cause immunosuppression for a long time. Extracellular vesicles derived from mesenchymal stem cells (MSC-EVs) prevented fibrosis in a sclerodermatous chronic GvHD mouse model by suppressing the activation of macrophages and the B cell immune response (122). The application of MSC-derived exosomes in GvHD and transplantation is summarized in Table 2.

**Table 1:** The application of MSCs in GvHD.

**Clinical research**	**Source of MSCs**	**Injection-method**	**Injection dose**	**Type of Study**	**Research results**	**References**
CBT combined MSCs (MSC-CBT)	BM-MSCs	Intramedullary injection	/	Phase I trial	Co-transplantation of MSCs may prevent GvHD with no inhibition of engraftment	(113)
CBT combined MSCs (MSC-CBT)	BM-MSCs	Intramedullary injection	0.5 × 10^6^/kg	Phase I trial	The safety of CBT combined with intrabone marrow injection of MSCs	(114)
GvHD after HSC transplantation	AT-MSCs	/	1 × 10^6^/kg, 3 × 10^6^/kg	Phase I/II trial	AT-MSCs, in combination with immunosuppressive therapy, may be considered feasible and safe	(115)
Akt1-MSCs Ameliorates Acute Liver GVHD.	BM-MSCs	/	/	Prospective controlled study	BM-MSCs genetically modified with Akt1 have a survival advantage and an enhanced immunomodulatory function	(116)
Steroid-refractory GvHD after HSC transplantation	BM-MSCs	Intravenous injection	6.81 × 10^6^/kg (range, 0.98–29.78 × 10^6^/kg)	Multi-center retrospective study	This therapeutic modality is safe and should be considered for steroid-refractory aGvHD	(117)

*CBT, cord blood transplantation; MSCs-CBT, CBT combined MSCs; HSC, haploid hematopoietic stem cell; AT-MSCs, adipose tissue-derived mesenchymal stromal cells; Akt1-MSCs, BM-MSCs genetically modified with AKT1; aGvHD, acute graft-versus-host disease*.

**Table 2:** The application of MSC-derived exosomes in GvHD and transplantation.

**Clinical research**	**Source of exo**	**Injection-methods**	**Injection dose**	**Type of Study**	**Research results**	**References**
An cGVHD mouse model	MSCs-exo/Fib-exo	Tail vein injection	Once a week for 6 weeks	Preclinical studies	MSCs-exo could improve the survival and ameliorate the pathologic damage of cGVHD by suppressing Th17 cells and inducing Treg	(120)
MSC-ex secreted by MSCs stimulated by different cytokines	huc-MSCs	/	/	Preclinical studies	TGF-β combined with IFN-γ exosome more effectively promoted the transformation of mononuclear cells to Tregs, IDO may play an important role	(54)
An aGVHD mouse model	BM-MSCs	Intravenous injection	/	Preclinical studies	The amelioration of aGVHD by therapeutic infusion of BM-MSC-derived EVs is associated with the preservation of circulating naive T cells	(123)
The lethal chimeric human-SCID mouse model of GvHD	MSCs-exo were incubated with mouse spleen CD4^+^ T cells	/	/	Preclinical studies	MSC exosome enhanced Treg production *in vitro* and *in vivo* through an APC-mediated pathway.	(121)
aGVHD	MSCs-exo	Intravenous injection	4 × 10^7^ MSCs was calculated as 1 unit, administered every 2–3 days until 4 units	Preclinical studies	MSC-derived exosomes may provide a potential new and safe tool to treat therapy-refractory GvHD	(119)
A mouse hepatic I/R model	MSCs-Heps-exo	Tail vein injection	100 μg	Preclinical studies	*In vivo*, MSC-Heps-Exo effectively relieve hepatic I/R damage, reduce hepatocyte apoptosis	(86)
A mouse hepatic I/R model	UC-MSCs	Tail vein injection	/	Preclinical studies	MiR-20a-containing exosomes from umbilical cord mesenchymal stem cells alleviates liver ischemia/reperfusion injury	(85)
Mouse models of CCl_4_-induced ALI/CLI	hucMSCs-exo	Tail vein injection	6 × 10^10^ particles/kg, 1.2 × 10^11^ particles/kg, 2.4 × 10^11^ particles/kg	Preclinical studies	hucMSC-Ex alleviated CCl4-induced acute liver injury and liver fibrosis and restrained the growth of liver tumors	(70)
Myocardial I/R model	MSCs-exo	Intramyocardial injection	50 μg	Preclinical studies	MSCs-exo attenuated myocardial I/R injury in mice via shuttling miR-182, which modified the polarization status of macrophages	(83)
Rats heart transplants model	IDO-BMSCs	Intravenous injection	800 mg/ml	Preclinical studies	Exosomes derived from IDO-BMSCs can be used to promote immunotolerance and prolong the survival of cardiac allografts	(97)
An rat IRI model	BMSCs-exo/Fib-exo	Intravenous injection	/	Preclinical studies	Rat BM-MSC-derived exosome protects against ischemia reperfusion injury with decreased inflammatory response and apoptosis in rats.	(84)
An IRR-induced ALL model	BMSCs-exo	Intravenous injection	5–10 μg	Preclinical studies	MSC-derived exosomes provide protection similar to that of MSCs against IIR-induced ALI via inhibition of TLR4/NF-κB signaling	(87)

*exo, exosome; cGVHD, chronic Graft-Versus-Host Disease; Fib-exo, exosomes from human dermal fibroblasts; huc-MSCs, human umbilical cord-derived MSCs; aGvHD, acute graft-versus-host Disease; SCID, severe combined immunodeficiency disease; I/R, Ischemia/Reperfusion injury; MSC-Heps-exo, mesenchymal stem cell-derived hepatocyte-like cell exosomes; CCl_4_, carbon tetrachloride; ALI, acute liver injury; CLI, chronic liver injury; IDO, indoleamine 2;3-dioxygenase; ALI, acute lung injury; IIR, intestinal ischemia-reperfusion*.

## Gene Editing of MSC-Derived Exosomes

MSC-derived exosomes have been confirmed to play a therapeutic role in a variety of disease models. If a method can be found for enhancing the efficacy of exosomes before infusion therapy, for example (e.g., gene-editing, tumor targeted delivery), it may expand the numbers of patients in a good prognosis (Figure 1) (124, 125). Different sources of MSCs affect the characteristics of the secreted exosomes; however, the MSCs are changed by changes in the external microenvironment. Can pretreatment with MSCs enhance the activity of exosomes to increase their efficacy? Studies have found that under a hypoxic environment, and after some cytokines and chemicals change the state of MSCs, their immune regulation function, including immune suppression and the ability to promote tissue and blood vessel regeneration, change (126). Under a hypoxic environment, the proliferation ability of BM-MSCs decreases, while adipose mesenchymal stem cells (AD-MSCs) increase. The gene and cell surface of MSCs can also be modified to enhance the therapeutic effect of exosomes. It has been reported that directly or indirectly increasing the activity of exosome pretreatment may be used to maximize the therapeutic potential of MSC-derived exosomes. For example, Ma et al. found that exosomes released by Akt-overexpressing MSCs showed beneficial effects in cardioprotection and angiogenesis, and the cardiac function of animals treated with Akt-Exo was significantly improved. The expression of platelet-derived growth factor D (PDGF-D) in Akt-Exo was significantly upregulated. In addition, Akt-Exo also significantly promotes the proliferation and migration of vascular endothelial cells for the formation of the tubular structure of blood vessels, tube-like structure formation *in vitro* and blood vessel formation *in vivo* (67). Regarding the application in the field of organ transplantation, Wen et al. modified human bone marrow mesenchymal stem cells so that their overexpression could inhibit the expression of fatty acid synthetase (Fas) interfering RNA and inhibit the expression of miR-375 RNA. The source and culture conditions of MSCs affected the function of exosomes. Exosomes co-cultured with PBMCs were transferred into an rat model of islet transplantation, downregulated Fas and miR-375 of islets. Then, by inhibiting the proliferation of PBMCs and enhancing Treg cell function, it exhibits an immunoregulatory function that significantly improves immune tolerance after pancreatic islet transplantation (127, 128).

Given the ability of MSCs to return to the nest site of injury, they may serve as a good carrier for MSC-derived exosomes. In order to increase the effect of exosomes, we aim to explore the exosomes synthesis/secretion pathway of MSCs. By gene editing or changing the microenvironment of MSCs, we hope to increase the number and purity of exosomes after pretreatment of MSCs, which can deliver accurate and large amounts of MSC-derived exosomes to target tissues effect. For example, we can preserve the original function of anti-inflammatory and tissue repair of exosomes. At the same time, we edit and introduce shRNA or miRNA with disease treatment effects into MSC-derived exosomes, so that exosomes could increase the gene therapy effect of specific nucleic acids. The author believes that in future clinical applications, especially for chronic rejection after organ transplantation, it will be possible to use MSC-derived exosomes to induce transplant tolerance. The edited pretreatment of MSC-derived exosomes plays an immunomodulatory role, which is expected to become a new direction in regenerative medicine and transplantation.

## Dose Issues and Commercial Production of MSC-Derived Exosomes

The dose dependence of MSCs and its exosomes is a difficult problem in clinical applications. At this stage, the main route of administration is intravenous injection, and most pre-clinical studies and clinical trials have used different doses. For example, one study showed that the intravenous administration of 1–3 × 10^6^/kg units of MSCs may reverse GvHD. In malignant tumors, 4 × 10^8^/kg could inhibit tumor growth, while low doses accelerated tumor proliferation (129). In a clinical study in novel coronavirus patients with pneumonia, 15 mL of exosomes derived from allogeneic bone marrow mesenchymal stem cells was added to 100 ml of normal saline, and administered intravenously 60 min. After a single intravenous injection of exogenous bone marrow exosomes, hypoxia, cytokines and immune reconstitution storms were profound reversal, and no patients developed adverse reactions in association with the treatment (130). However, the biological role of exosomes is not yet fully understood, and we still need to pay close attention to their side effects. It is certain that MSC-derived exosomes play an immunomodulatory role, and we are confident that the research of MSC-derived exosomes will make a major breakthrough in the future.

For clinical safety, the production of MSCs for therapeutic purposes must comply with good manufacturing practices to ensure the provision of safe, repeatable and efficient products. The production of safe and reliable clinical MSCs includes multiple requirements, including tissue sources, culture methods, and quality control standards. It is now believed that exosomes have low immunogenicity and tumorigenicity. In comparison to simple MSCs, MSC-derived exosomes are well-tolerated. And they are more convenient and practical to be applied *in vivo*. Thus, MSC-derived exosomes have broad prospects as a therapeutic agent. It is worth noting that although MSC exosomes avoid the risks of immunogenicity and tumorigenicity that are associated with cell transplantation, there are still many problems associated with application in the clinical setting, including production standards (e.g., culture separation, cell phenotype, quantification, and positioning tracking after infusion) and the monitoring of efficacy (131). The determination of clinical indications for different diseases, especially the production of standard dosage in the field of organ transplantation, is a problem that needs to be solved urgently.

**Figure 2:**
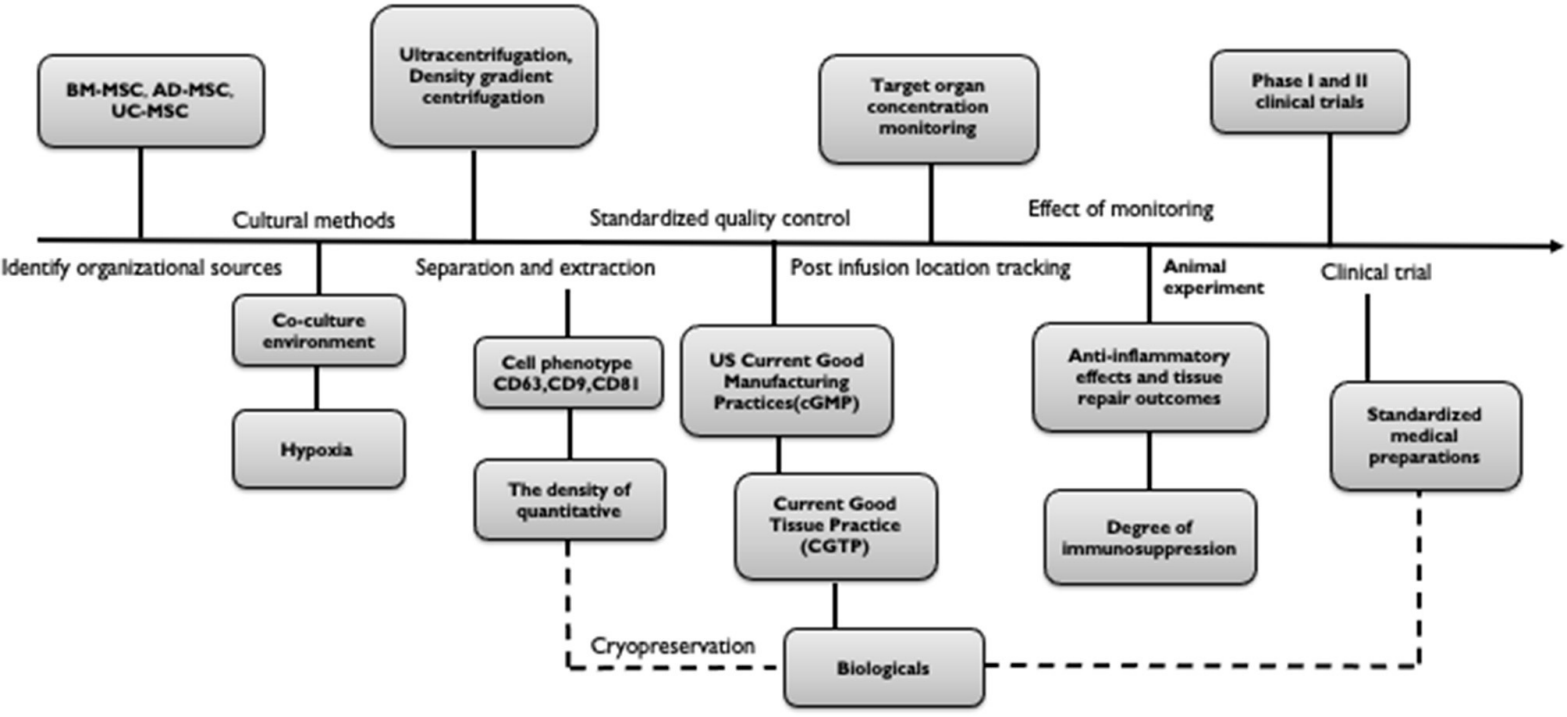
Standardization process for the preparation of exosomes for medical biologics. The process is as follows: (a) Identify organizational sources (BM-MSC, AD-MSC, UC-MSC, etc.). (b) Culture and separation (condition, environment, segregation pattern). (c) Test confirmation (cell phenotype, quantity, purity). (d) Standardized quality control (cGMP, CGTP). (e) Post-infusion location tracking (target organ concentration monitoring, efficacy monitoring). (f) Clinical trial (Phase I and II).

Studies have suggested that for the actual production of therapeutic exosomes, some lentiviral vectors that are currently being tested in clinical trials should be used to transform cells. It is extremely important to establish methods that MSC-derived exosomes can be stably supplied. We not only aim to expand cell phenotypes of MSC-derived exosomes but also to pay attention to not damage the original roles of the MSC at the same time of increasing the production of exosomes. Thus, the optimization of cryopreservation strategies for the long-term storage of functional MSC-derived exosomes may be helpful for the development of off-the-shelf biologics (132). Current methods that are important for the extraction of exosomes are ultracentrifugation and density gradient centrifugation. However, methods for obtaining exosomes with high- quantity and high-purity remain a focus of research. Cytokine priming upregulated RAB27B siRNA in AD-MSCs, increasing their secretion of exosomes, which is a useful strategy for harvesting anti-inflammatory MSC-EVs for clinical applications (39). As mentioned earlier, gene editing is used to overexpress therapeutically effective molecules (such as mRNA, miRNA, and proteins) in MSCs to produce characteristic MSC-derived exosomes for different diseases. Alternatively, the therapeutic efficacy may be increased by targeting organ-targeted MSC-derived exosomes. In addition to the production standards in biomedical engineering, it is equally important to verify the safety and feasibility through a large number of pre-clinical studies and clinical trials. The specific phenotype and function and the degree of conversion should be verified by quality control methods and should adhere to quality standards. In this section, we summarized the preparation of a standardization process for the medical application of exosomes as biologic agents (Figure 2). However, the standard has not yet reached a consensus. Although we have put forward some points, we still need a multi-center study to determine a feasible solution.

## Concluding Remarks

We hope that MSC-derived exosomes research will open up new medical and pharmaceutical fields while focusing on safety, the standardization of production steps, and determination of therapeutic doses, providing new viable commercial products for the treatment of organ transplantation and GvHD, and other diseases.

## Author Contributions

QYZ, SJZ, W-ZG, and X-KL conceived and designed the manuscript. QYZ and X-KL wrote the paper. All authors listed have made a substantial, direct and intellectual contribution to the work, and approved it for publication.

## Conflict of Interest

The authors declare that the research was conducted in the absence of any commercial or financial relationships that could be construed as a potential conflict of interest.
